# β-Radiation Stress Responses on Growth and Antioxidative Defense System in Plants: A Study with Strontium-90 in *Lemna minor*

**DOI:** 10.3390/ijms160715309

**Published:** 2015-07-07

**Authors:** Arne Van Hoeck, Nele Horemans, May Van Hees, Robin Nauts, Dries Knapen, Hildegarde Vandenhove, Ronny Blust

**Affiliations:** 1 SCK•CEN, Belgian Nuclear Research Centre, Boeretang 200, 2400 Mol, Belgium; E-Mails: nhoreman@sckcen.be (N.H.); mvhees@sckcen.be (M.V.H.); rnauts@sckcen.be (R.N.); hvandenh@sckcen.be (H.V.); 2 Department of Biology, University of Antwerp, Groenenborgerlaan 171, 2020 Antwerpen, Belgium; E-Mail: ronny.blust@uantwerpen.be; 3 Centre for Environmental Research, University of Hasselt, Universiteitslaan 1, 3590 Diepenbeek, Belgium; 4 Veterinary Sciences, University of Antwerp, Groenenborgerlaan 171, 2020 Antwerpen, Belgium; E-Mail: dries.knapen@uantwerpen.be

**Keywords:** radiation responses, abiotic stress, dosimetry, *Lemna minor*, oxidative stress, strontium-90

## Abstract

In the following study, dose dependent effects on growth and oxidative stress induced by β-radiation were examined to gain better insights in the mode of action of β-radiation induced stress in plant species. Radiostrontium (^90^Sr) was used to test for β-radiation induced responses in the freshwater macrophyte *Lemna minor*. The accumulation pattern of ^90^Sr was examined for *L. minor* root and fronds separately over a seven-day time period and was subsequently used in a dynamic dosimetric model to calculate β-radiation dose rates. Exposing *L. minor* plants for seven days to a ^90^Sr activity concentration of 25 up to 25,000 kBq·L^−1^ resulted in a dose rate between 0.084 ± 0.004 and 97 ± 8 mGy·h^−1^. After seven days of exposure, root fresh weight showed a dose dependent decrease starting from a dose rate of 9.4 ± 0.5 mGy·h^−1^. Based on these data, an EDR_10_ value of 1.5 ± 0.4 mGy·h^−1^ was estimated for root fresh weight and 52 ± 17 mGy·h^−1^ for frond fresh weight. Different antioxidative enzymes and metabolites were further examined to analyze if β-radiation induces oxidative stress in *L. minor*.

## 1. Introduction

Anthropogenic activities have led to enhanced releases of fission products from nuclear facilities into the environment. Many of these fission products are radionuclides that are short-lived γ- and β-emitting isotopes, which rapidly lose their radioactivity. However, some radionuclides have a physical half-life that takes several years and therefore can contribute to a long-lasting contamination of the biosphere [1]. Among the artificially produced radionuclides, strontium-90 (^90^Sr) is of particular interest due to its relative long physical half-live of 28.6 years. It is produced with high yields as a byproduct of the fission of uranium and plutonium and is thus a common waste product from nuclear activities [2]. Also, the explosions of nuclear weapons in the 1950s and 1960s contributed to the widespread distribution of ^90^Sr in the environment [3]. More recently, ^90^Sr was accidently released during nuclear meltdown in Fukushima in 2011 [4] and in substantial amounts in Chernobyl in 1986 [5]. Geochemical studies revealed that large amount of this radionuclide can be transported via rivers to the ocean or could accumulate in freshwater reservoirs where it can remain for many years [4,6]. As ^90^Sr decays, it releases moderate energy β-particles and forms yttrium-90, which in turn emits very strong energetic β-particles within 64 h to stable zirconium. Since most of the energy from such β-particles is absorbed by surface tissues, the bioaccumulation of these radionuclides can potentially produce deleterious effects to the health of living organisms [1].

Radioecological research on β-emitting isotopes in plants has mainly focused on ^90^Sr accumulation in edible plants, in particular crops [7,8,9,10] or on exploring the use of plants in phytoremediation strategies *i.e.*, to decontaminate soils contaminated with low levels of ^90^Sr [11,12,13,14]. A number of studies report on a reduction in plant growth at elevated Sr concentrations [10,15,16,17]. However, these focused on stable Sr at the mM-level. Biological responses induced by β-radiation from ^90^Sr have, to date, not been studied in detail in plants [18]. Only one study examined the photosynthetic response induced by high ^90^Sr exposure levels [19]. Relative biological effectiveness (RBE) values can be used to extrapolate the biological responses for different types of radiation in non-human biota. Since RBE values for high-energy β-radiation are equal to γ-radiation, similar radiation responses can be expected compared to γ-radiation exposure [20]. A transcriptomic study revealed similar stress responses, defense responses and metabolic processes of rice seedlings exposed differentially to either γ-radiation or particle radiation [21]. High levels of ionizing radiation often lead to an increase in the formation of highly reactive oxygen species (ROS), which may cause oxidative stress, a disturbance of the cellular redox status, as is observed after exposure to other environmental stressors [22,23]. These ROS molecules are also generated as byproducts of normal oxidative metabolism and play an imported role in signaling and oxidative stress responses. Under abiotic stress, an imbalance between oxidative and reductive processes in the cell is generated. Plants have antioxidative defense systems to counteract the elevated ROS levels. These comprise of low molecular weight antioxidants like reduced glutathione (GSH) and ascorbate (ASC). The metabolites are key players in the ASC-GSH cycle, forming an important mechanism of the antioxidative defense system [24]. Other antioxidative biomolecules include antioxidative enzymes such as catalase (CAT), glutathione reductase (GR), superoxide dismutase (SOD), ascorbate peroxidase (APOD), syringaldizyne peroxidase (SPOD) and guaiacol peroxidase (GPOD). Together these enzymatic and non-enzymatic antioxidants can scavenge different free radical elements in order to maintain the redox balance in the cell and to protect biomolecules from oxidative attacks. Different studies already highlighted the importance of such antioxidative enzymes and metabolites to handle γ-radiation exposure treatment in plants [25,26]. However, antioxidative defense mechanisms have never been examined in β-irradiated plants.

Although Sr is not essential for plant metabolism, it bears a chemical analogy with the essential plant macronutrient Ca. In terrestrial plants, the uptake of Ca occurs mainly via Ca channels in the roots [27]. Calcium ATPases are also present in the roots and catalyze the Ca influx and efflux across the plasma membrane of endodermal cells. Calcium is then transported to the shoots via the xylem where the excess of Ca is stored in vacuoles to keep the Ca concentration in cytosol at a basal level. Kinetic studies of Ca in combination with Sr pointed out that both compounds have almost identical uptake efficiencies [11,17,28]. Furthermore, it has been found that once taken up, like Ca, Sr is exclusively transported via the xylem, which further led to the conclusion that plants do not discriminate Sr and Ca ions [29,30]. Calcium oxalate crystals are formed at toxic Ca concentration in plants and when Sr is added to nutrient solutions, it has been found that Sr is incorporated in Ca oxalate crystals in *L. minor* [31,32]. Overall, plants seem to have the capacity to accumulate Sr-ions, thereby taking advantage of Ca uptake and transport mechanisms. Therefore, it is conceivable that when the radioactive isotope ^90^Sr accumulates, it can subsequently deliver a considerable β-radiation dose to the plant organs.

*Lemna minor* was selected as a biological model to study irradiation effects following β-radiation exposure. This free-floating vascular macrophyte develops roots and has the potential to take up nutrients through both fronds and roots. *L. minor* is to date the only freshwater plant model for testing metal biomonitoring and other toxic chemicals in freshwater ecosystems [33,34]. This bio-test includes a seven-day experiment to evaluate growth related endpoints. This study reports on the responses of *L. minor* to β-radiation by using ^90^Sr as β-emitting isotope. First, the uptake pattern of ^90^Sr was characterized over a seven-day time period to construct a dynamic dosimetric model to determine internal and external exposures rates. Afterwards, *L. minor* plants were exposed to different ^90^Sr concentrations in order to study possible dose dependent relationships. The classical growth related endpoints were evaluated and compared with antioxidative defense system endpoints. Our findings contribute to a better insight into the antioxidant defense system in plants following β-radiation. 

## 2. Results

### 2.1. ^90^Sr Uptake and Dosimetry

**Figure 1:**
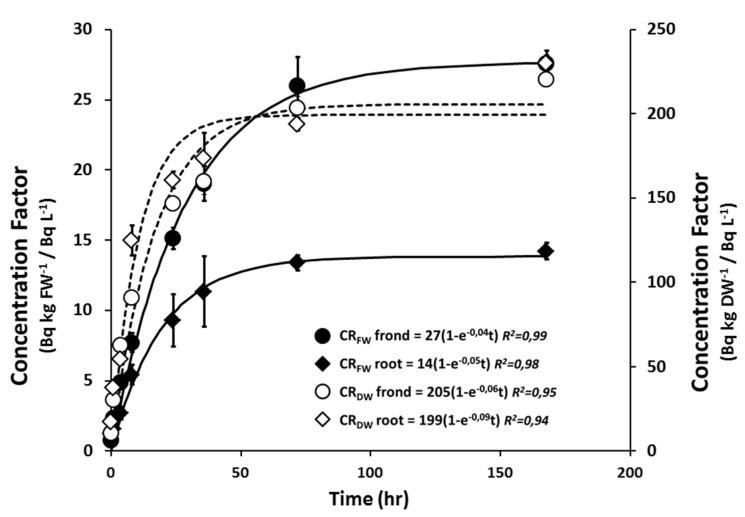
Time course of Sr^90^ uptake in *L. minor* for time period of 168 hours exposed to 10 kBq·L^−1^ of ^90^Sr. Black dots refers to concentration ratios on fresh weight for frond and root, white dots refers to concentration ratios on dry weight for frond and root. Legend shows the equation of each fit with the *R*^2^ value. Each data point represents the mean ± SE of six biological replicas.

*L. minor* plants were continuously exposed for seven days to four different activity concentrations from 25 up to 25,000 kBq·L^−1^ to study dose dependent relationships of β-radiation. In order to constitute a maximal ^90^Sr uptake and accumulation, *L. minor* plants were exposed in a medium containing 0.2 mM Ca. The ^90^Sr concentration ratios (CR) to frond and root expressed on fresh and dry weigh basis are shown in Table 1. Although the CR_DW_ of the highest tested activity concentration was slightly lower compared with other tested activity concentrations, no significant differences in CR_DW_ were observed between tested Sr activity concentrations from non-exposed plants (ANOVA, *p* < 0.05). It was found that the ^90^Sr content was similar in both plant organs (fronds and roots) indicated by equivalent CR_DW_ for each tested activity concentration. In contrast, the CRs expressed on plant fresh weight (CR_FW_) and used in dosimetry calculations, were remarkably lower in the roots compared to the fronds. The CR_FW_ in this study were approximately 60 and 30 Bq·kg^−1^ FW/Bq·L^−1^ for fronds and roots respectively. As for CR_DW_, no concentration-dependent differences in CRs were observed for the tested activity concentrations. ^90^Sr uptake in *L. minor* roots and fronds was monitored over a seven-day period in order to enable more correct dose assessment estimations. With the tested ^90^Sr activity concentration used in this experiment, the activity of ^90^Sr found in roots increased rapidly during the first 8 h, then much slower during the next 72 h before it tended to stabilize (Figure 1). About 92% ± 3% of the ^90^Sr uptake capacity at equilibrium phase (at which the net uptake is zero) of the *L. minor* fronds and 97% ± 4% of the roots was accumulated after 72 h of exposure. These time-dependent uptake parameters and the respective CR_FW_ were considered to calculate accurate dose rates for *L. minor* organs (fronds and roots) exposed over a seven-day exposure. Calculating internal and external dose rates for roots and fronds separately, the exposure of *L. minor* plants to ^90^Sr activity concentration from 25 up to 25,000 kBq·L^−1^ resulted in a total dose rate ranging from 0.084 ± 0.004 and 97 ± 8 mGy·h^−1^ for the *L. minor* plants (Table 1). The estimated absorbed dose rates from internal exposure were generally one order of magnitude higher than those from external exposure.

**Table 1:** Nominal and measured medium activity concentrations of ^90^Sr, biomass of *L. minor* fronds and roots given as fresh weight (FW) and dry weight (DW) of each activity concentration, ^90^Sr concentration ratios expressed in fresh weights and dry weights for *L. minor* fronds and roots after seven days of exposure and the calculated absorbed dose rates in *L. minor* organs. All values are given as mean ± SE with at least three biological replicates. Significant differences from control plants (*p* < 0.05, one-way ANOVA) are given as *.

Activity Concentration			Biomass		Uptake Parameters		Dosimetric Parameters		
Nominal [kBq·L^−1^]	Measured [kBq·L^−1^]		FW [mg]	DW [mg]	Conc. Ratio FW [Bq·kg^−1^ FW/Bq·L^−1^]	Conc. Ratio DW [Bq·kg^−1^ DW/Bq·L^−1^]	Dose Rate Intern [µGy·h^−1^]	Dose Rate Extern [µGy·h^−1^]	Total Dose Rate [µGy·h^−1^]
0	0 ± 0.001	plant	199.5 ± 10	13.9 ± 0.7	na	na	na	na	na
		frond	165.6 ± 7.8	12.3 ± 0.6	na	na	na	na	na
		root	33.8 ± 2.3	1.6 ± 0.1	na	na	na	na	na
25	25 ± 0.1	plant	225.3 ± 10.4	15.3 ± 0.5	60 ± 3	832 ± 21	85 ± 4	5 ± 0.2	84 ± 4
		frond	186 ± 8.8	13.5 ± 0.3	62 ± 2	829 ± 30	86 ± 4	5 ± 0.2	89 ± 4
		root	38.8 ± 1.8	1.8 ± 0.2	28 ± 2	783 ± 23	41 ± 3	10 ± 0.4	51 ± 2
250	247 ± 0.1	plant	210.2 ± 9.6	15.1 ± 0.9	60 ± 4	767 ± 65	918 ± 63	53 ± 3	967 ± 58
		frond	178 ± 8	13.7 ± 0.7	65 ± 5	789 ± 71	1000 ± 76	53 ± 3	1053 ± 74
		root	31.2 ± 1.6	1.5 ± 0.1	29 ± 1	752 ± 15	454 ± 20	107 ± 5	562 ± 21
2500	2453 ± 2	plant	195.6 ± 5.6	14.0 ± 0.2	60 ± 3	844 ± 23	8881 ± 486	530 ± 3	9432 ± 483
		frond	178.5 ± 5	13.2 ± 0.3	63 ± 3	858 ± 24	9185 ± 477	530 ± 3	9715 ± 476
		root	16.8 ± 0.7 *	0.9 ± 0.1 *	37 ± 3	820 ± 48	5486 ± 469	1070 ± 6	6556 ± 464
25,000	24,567 ± 13	plant	124.4 ± 3.4	12.5 ± 0.1	57 ± 5	540 ± 35 *	91,063 ± 8607	6006 ± 98	97,264 ± 8490
		frond	118.7 ± 3.5 *	12.2 ± 0.1	59 ± 6	542 ± 36 *	92,827 ± 9130	6006 ± 98	98,834 ± 9033
		root	5.7 ± 0.2 *	0.4 ± 0.02 *	36 ± 2	474 ± 20 *	56,904 ± 2287	12,121 ± 197	69,025 ± 2181

### 2.2. ^90^Sr Induced Effects in L. minor

**Figure 2:**
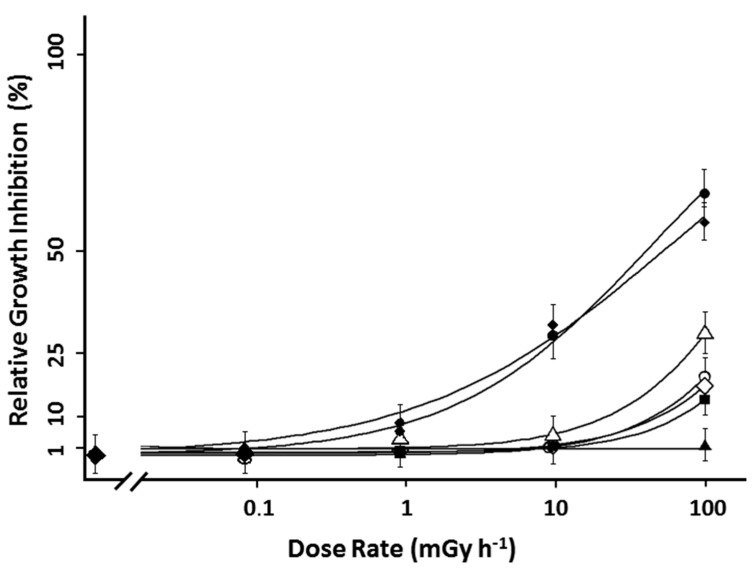
Dose response curve of *L. minor* plants treated for seven days with β-radation. The dose response curces are given by relative growth inhibition based on frond area (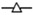), frond number (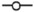), plant fresh weight (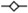), frond fresh weight (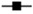), frond dry weight (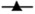), root fresh weight (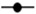) and root dry weight (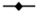). Each data point represents the mean ± SE of nine biological replicas for frond related endpoints and three biological replicas for root related endpoints. In addition to the average values of each point, the log-logistic function fitted through the data is added.

Frond number, frond area and frond and root fresh and dry weight were measured for *L. minor* plants exposed from 0.084 ± 0.004 mGy·h^−1^ up to 97 ± 8 mGy·h^−1^. After seven days, frond related growth rates determined based on fresh weight, frond area and number decreased significantly at the highest tested dose rate of 97 ± 8 mGy·h^−1^ with an inhibition of 16% ± 3%, 18% ± 1% and 16% ± 3%, respectively (ANOVA, *p* < 0.05). The growth endpoints measured on root fresh and dry weight were significantly affected at 9.4 ± 0.5 mGy·h^−1^. At the highest tested dose rate, root fresh and dry weight was inhibited for 63% ± 1.5% and 57% ± 0.4%, respectively (Figure 2). The derived EDR_10_-value (Effective Dose Rate) for frond area was 95 ± 7, and 154 ± 15 and 152 ± 13 mGy·h^−1^ for fresh weight and frond number, respectively. The EDR_10_-values for root biomass were 1.5 ± 0.4 and 1.0 ± 0.5 mGy·h^−1^ for root fresh and dry weight, respectively. Growing *L. minor* plants in stable Sr concentrations up to 60 nM, the equivalent nominal concentration at ^90^Sr activity concentration of 25,000 kBq·L^−1^, did not result in a significant difference in growth rate (data not shown). As such, observed growth effects are expected to be linked to ^90^Sr induced radiotoxicity and not Sr chemotoxicity.

**Figure 3:**
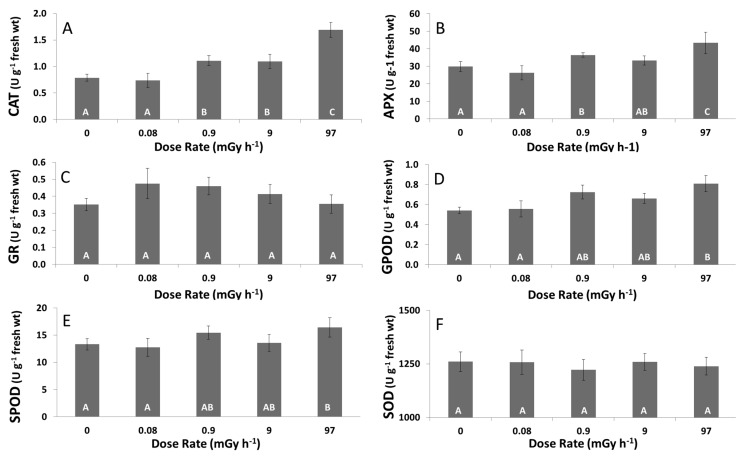
Enzyme activities of (**A**) catalase (CAT); (**B**) ascorbate peroxidase (APX); (**C**) glutathione reductase (GR); (**D**) guaiacol type peroxidase (GPOD); (**E**) syringaldizine peroxidase (SPOD); and (**F**) superoxide dismutase (SOD) in *L. minor* exposed to different dose rates levels of β-radation for seven days. Each data point represents the mean ± SE of three biological replicas. Different capital letters indicate significant differences between treated plants and control plants (*p* < 0.05, one-way ANOVA).

To evaluate oxidative stress induced in β-exposed *L. minor* plants, antioxidative enzyme activities including CAT, GR, SOD, APX, GPOD and SPOD were analyzed (Figure 3). No significant differences in enzyme activities were found in plants exposed to the lowest dose rate at 0.084 ± 0.004 mGy·h^−1^. At the next tested dose rate 0.97 ± 0.06 mGy·h^−1^, enzyme activities of CAT and APX were significantly enhanced, which remained status quo at the next tested dose rate. At the highest dose rate tested, a significant increase in CAT, APX, GPOD and SPOD activities in comparison to non-treated plants was observed. At the dose rate of 97 ± 8 mGy·h^−1^, an approximately twofold increase in activity was evidenced for CAT, while enhancement in activity was less pronounced for the other enzymes. GR and SOD were the only enzymes exhibiting no alterations in activity with increasing dose rate.

**Figure 4:**
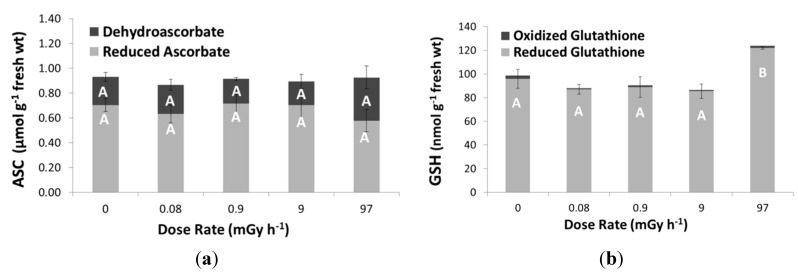
Metabolite concentrations of (**a**) ascorbate (ASC) and (**b**) glutathione (GSH) in *L. minor* exposed to different dose rates levels of β-radation for seven days. Concentration levels of reduced and oxidized forms of the metabolites are shown in light and dark grey bars, respectively. Each data point represents the mean ± SE of three biological replicas. Different capital letters indicate significant differences between treated plants and control plants (*p* < 0.05, one-way ANOVA).

Concentrations of oxidized and reduced forms of ASC and GSH were determined to study their redox status (Figure 4). No significant shift in redox status was observed at any dose rate intensity for ASC. In contrast, an increase in GSH content was observed at the highest tested dose rate of 97 ± 8 mGy·h^−1^. At this dose rate, the total GSH concentration attained a level of 121± 1 nmol·g^−1^ FW while control plants exhibited a GSH concentration of 96 ± 8 nmol·g^−1^ FW.

## 3. Discussion

### 3.1. ^90^Sr Accumulation and Uptake

The present study aimed at evaluating β-radiation induced growth and oxidative stress responses in *L. minor* to a range of ^90^Sr exposure concentrations. Therefore, *L. minor* plants were exposed above the threshold level of 400 µGy·h^−1^ since dose rates under this level are considered not to induce detrimental effects to some individuals in aquatic organisms [35]. Exposure media were adjusted with low Ca concentrations to induce high ^90^Sr uptake in *L. minor* plants and subsequent high internal β-radiation exposure. The time course of ^90^Sr uptake showed that the ^90^Sr content of *L. minor* roots and fronds tended to saturate over time. The ^90^Sr uptake reached a plateau after three days in both roots and shoots, indicating that ^90^Sr concentration in plants was in equilibrium with the ^90^Sr concentration of the growth medium. Similar results were obtained with Sr uptake in large-flowered waterweed and in hydroponically grown maize and sunflower where a steady state for ^90^Sr/Sr uptake between was obtained after three to five days [17,36,37]. The CR_FW_ in present study were approximately 60 and 30 Bq·kg^−1^ FW/Bq·L^−1^ for fronds and roots, respectively. These are among the lower CR_FW_ compared to other floating-leafed macrophophytes [38,39,40,41]. Phylogenetic studies suggest that monocotyls, like *L. minor*, have low capacity for Ca accumulation [11]. As it is generally presumed that Sr-ions are homologous to Ca ions and thus behave like Ca in organisms, low accumulation capacity of Sr is possibly linked with low Ca content in plants biomass.

### 3.2. Dosimetry

To obtain accurate dose rate estimates, *L. minor* was split in two functional units (fronds and roots) each having tissue-specific dose conversion coefficients. The calculated total dose ranged between 0.084 ± 0.004 mGy·h^−1^ up to 97 ± 8 mGy·h^−1^, of which the lowest dose rate was of the same order of magnitude as the IAEA threshold dose rate. After seven days of exposure, the second highest dose rate tested (9.4 ± 0.5 mGy·h^−1^) had a negative impact on root growth. According to the EDR values, the root related endpoints were about twenty times more sensitive compared to frond related endpoints. Highly sensitive stress responses on *L. minor* root growth were also observed in metal and pesticide toxicity. Following this, *L. minor* root growth/length has been suggested as an alternative bioassay for toxicity testing [42,43,44]. All endpoints, including frond biomass, frond number and frond area, showed a clear decrease at the highest tested dose rate (97 ± 8 mGy·h^−1^). From the available literature dealing with effect studies [45,46], no reports were found on ^90^Sr or β-radiation induced effects in aquatic plants, emphasizing a large data gap for aquatic plants [35,45]. However, daphnids exposed to a dose rate of 1.8 mGy·h^−1^ with ^90^Sr revealed a 36% reduction in life span, while another study with daphnids using ^90^Sr, showed a decrease of 26% in fertility rate after exposure to 0.8 mGy·h^−1^ [45,47]. These dose rates at which growth effects on these aquatic invertebrates were observed are comparable to the dose rates at which *L. minor* root growth was affected. Since β-particles are proposed to have an RBE factor of 1 (or 3 for low-energy β-radiation), it is generally accepted that radiological effects caused by γ-radiation can also be adopted for making radiation comparisons. From a 14-year field experiment in the Canadian Boreal forest, it has been found that the most sensitive of plant species studied, pine trees, died within 2–3 years when exposed to a dose rate of 5–10 mGy·h^−1^ and within two weeks at an estimated dose rate of 250–300 mGy·h^−1^ for pine trees close to the damaged reactor in Chernobyl [48,49]. In the present study, EDR_50_ values ranged between 250 and 500 mGy·h^−1^ for *L. minor* fronds, suggesting that *L. minor* is more radioresistant than pine trees. Garnier-Laplace, *et al.* [50] established a species sensitivity distribution curve for terrestrial plants chronically exposed to γ-radiation under laboratory and field conditions and derived EDR_10_ values on population related endpoints. Considering the most sensitive EDR_10_ endpoint obtained in this study (1.0 ± 0.5 mGy·h^−1^ for root dry weight), approximately 50% of the selected organisms from the SSD curve were more radioresistant in comparison to *L. minor*.

### 3.3. Antioxidative Response

Oxidative stress related genes are among the most represented genes in responding to radiation in *Arabidopsis* plants [51,52]. Therefore, to gain more insights in the antioxidative stress response of *L. minor* plants following β-radiation exposure, some features of the ROS scavenging biosynthetic pathways involving both enzymatic and non-enzymatic processes were studied. There were minor changes in total ASC content or redox status, although an enhanced activity of APX was found. Increased APX activity with a stable ASC content was also found in other duckweed studies under chemical stress [53]. Under severe oxidative stress conditions, an enhanced APX activity is typically associated with a decrease in reduced ASC content to remove H_2_O_2_ from the cytosol and chloroplasts [54]. Similar, at the highest dose rate tested, the increase in APX activity followed by slight decrease in reduced ASC levels observed points to an increased oxidative stress under these severe radiation conditions.

The concentration of the other antioxidant studied, GSH, significantly increased at the highest tested dose rate of 97 mGy·h^−1^. An increased GSH content was also found in other plants tissues suffering from oxidative stress to compensate ascorbate oxidation [55,56,57]. The fact that redox status of GSH remained virtually unchanged could explain the unaltered GR activity since this enzyme is responsible for maintaining the GSSG/GSH ratio constant [22]. Also in acutely γ-irradiated red pepper plants (0–10 Gy), no changes were reported in GR activity upon increasing radiation dose [58]. However, the higher level of GSH and indications that ASA redox starts to decline at 97 ± 8 mGy·h^−1^ corroborate the hypothesis that *L. minor* plants have difficulty in controlling the production of ROS under high radiation stress. In addition to APX, also CAT increased at levels below the EDR_10_ value. Increased CAT activity is supposed to be an adaptive trait helping to reduce toxic levels of H_2_O_2_ in peroxisomes [54]. Sensitive responses on CAT activity were also reported for *S. capillata* exposed to 65 µGy·h^−1^ with γ-radiation. *L. minor* plants exposed to radiofrequency radiation and in *A. thaliana* where increased expression levels of CAT1 and CAT3 genes were observed at 50 mGy·h^−1^ [25,59,60]. Therefore, it does seem that plants rely on CAT as their first defense to counter radiation-mediated ROS production. On the contrary, SODs are regarded as the first line of defense against ROS under several abiotic stressors, but according to our findings, β-radiation did not induce any alterations in SOD enzyme activity [22,54]. For acutely γ-irradiated red pepper, a weak enhancement in SOD activity was found from 2 Gy, while SOD activity in chronically irradiated *Stipa capillata* (65 µGy·h^−1^) did not show any response [58,60]. Also, GPOD, GR, and SPOD were not or only slightly affected at the highest dose rate, which might indicate that a β-irradiation treatment of 97 ± 8 mGy·h^−1^ for seven days. However, since growth was clearly hampered at the highest dose rate, available energy supplies might be used for other metabolic pathways to neutralize β-radiation stress in *L. minor* plants.

## 4. Experimental Section

### 4.1. Culture Stock

*Lemna minor* cv. Blarney plants were obtained from Dr. M. Jansen (University College Cork, Cork, Ireland) and cultured aseptically in 250 mL glass Erlenmeyer flasks containing half-strength Hütner medium [34] under continuous light (Osram 400 W HQI-BT daylight, OSRAM GmbH, Augsburg, Germany, 80–100 µmol·m^−2^·s^−1^) at 24 °C. Plants were sub-cultured every 10–12 days by transferring three plants to 100 mL of fresh growth medium. To obtain sufficient and homogenous plant population, 1 week before the experiment a preculture was initiated with five mature plants per pot (3–4 fronds) in 100 mL of fresh medium.

### 4.2. ^90^Sr Exposure

For the ^90^Sr treatment, 3 healthy duckweed plants were chosen randomly and transferred to 250 mL PC containers (VWR) containing 25 mL experiment nutrient solution. For the ^90^Sr uptake experiments, modified K-medium (0.4 mM Ca and (in mg·L^−1^) 889 KNO_3_, 95 Ca(NO_3_)_2_·4H_2_O, 500 MgSO_4_·7H_2_O, 9 Na-EDTA, 3 tartaric acid, 1.86 H_3_BO_3_, 0.22 ZnSO_4_·7H_2_O, 0.12 Na_2_MoO_4_·2H_2_O, 0.08 CuSO_4_·5H_2_O, 3.62 MnCl_2_·4H_2_O, 5.4 FeCl_3_·6H_2_O with lower KH_2_PO_4_ concentrations (0.5 mg·L^−1^) [61]) was used containing an activity concentration of 10 kBq·L^−1^ ^90^Sr, added as SrCl_2_ (3.7 MBq stock solution, IDB Belgium) for 6 biological replicates. For the ^90^Sr effect experiments, a modified Steinberg medium (0.2 mM Ca and (in mg·L^−1^) 350 KNO_3_, 50 Ca(NO_3_)_2_·4H_2_O, 0.09 KH_2_PO_4_, 100 MgSO_4_·7H_2_O, 1.5 Na-EDTA, 0.12 H_3_BO_3_, 0.18 ZnSO_4_·7H_2_O, 0.044 Na_2_MoO_4_·2H_2_O, 0.18 MnCl_2_·4H_2_O, 0.76 FeCl_3_·6H_2_O [62]) was used having activity concentrations of 25, 250, 2500 and 25,000 kBq·L^−1^ ^90^Sr for 9 biological replicates. Since *L. minor* plants exposed in Steinberg medium had the highest Sr^90^ uptake, and hence highest dose rate, this medium was selected for all effect experiments. The growth of the plants did not differ between both exposure media. The chemical concentration of Sr at the highest tested activity level amounted to 54 nM. Plants were exposed in a growth chamber in sterile environment under continuous light (80 to 100 µmol/s m^2^) at 24 °C during ^90^Sr treatment. Plants were harvested after 7 days of exposure by washing in 1 mM Pb(NO_3_)_2_ and twice in distilled water for 10 min in order to discriminate ^90^Sr accumulation from ^90^Sr adsorption on root and frond surface. A significant difference was found between plants washed with Pb-solution compared to non-washed or washed with distilled water (*p* < 0.05, data not shown). On the other hand, it was confirmed that there was no significant difference in washing the *L. minor* plants with a solution containing an excess of Pb (1 mM) with plants washed in a high Ca-solution (10 mM. data not shown). Fresh weight was determined by weighing all collected plant material from all individual pots after quickly dry patting with clean tissue. For the ^90^Sr effect experiments, plants from 3 pots were further dried for 2 days at 60 °C (humidity < 5%) for dry weight determination and Sr uptake measurement. Plants from the remaining 6 pots were, after fresh weight determination, snap frozen in liquid nitrogen for storage at −80 °C for biochemical analysis.

### 4.3. ^90^Sr Transfer and Dosimetry

All collected plant material from 3 individual pots was dry-ashed in a muffle furnace, and subsequently digested in 0.1 M HCl. For ^90^Sr uptake experiments, all 6 individual pots were used. Digested samples were then diluted 10-fold in dH_2_O, vortexed and diluted again 4-fold in scintillation cocktail (Optiphase Hisafe 3, PerkinElmer). Samples (5 mL) were taken from each pot at the end of the experiment and brought to 20 mL with the same scintillation cocktail. After careful mixing of sample and scintillation fluid, the ^90^Sr activity was measured for 60 min by β-liquid scintillation counting (Packard 1600TR Tri-Carb, Canberra, Zellik, Belgium). The counting efficiency was determined using a dilution series of ^90^Sr with known activities. No difference was found between concentrations in samples from growth medium before and after the experimental period indicating that ^90^Sr was dissolved during the exposure period (data not shown). Concentration Ratios (CR) are expressed as the ratio of ^90^Sr activity in the plant organ (root and frond) (in Bq·kg^−1^) and the external solution ^90^Sr content (expressed in Bq·L^−1^). Concentration ratios were calculated on a organ fresh weight (CR_FW_) and organ dry weight (CR_DW_) basis. CRs were calculated for roots and fronds.

Dosimetry of radionuclides in species with small sizes requires special attention for accurate dose rate calculations since the range of high-energy β-radiation in biological matter exceeds the geometrical size of species. Therefore, *L. minor* plants were split in two functional units for dose calculations. The internal and external dose conversion coefficients (DCC, in µGy·h^−1^ per Bq·kg^−1^·FW) were calculated by the ERICA tool for root and shoot separately [39]. The estimated dimensions of fronds and roots necessary for use in ERICA tool were determined by picture analyses using the ImageJ open source software (version 1.43) [63]. An average control *L. minor* plant had the following dimensions: 0.25 mm thick, 3 mm wide and 3.5 mm deep for frond and the average root length determined at 90 mm long with a diameter of 0.15 mm. Within the Tier 2 assessment, DCCs can be calculated for any organism by entering the required radionuclide and the organism’s dimensions (the mass and the three axes that define the ellipsoid used as its simplified representation). The geometries for both organs were separately entered in the software and the DCCs obtained were in agreement with the DCCs obtained from Biermans *et al.* [19]. The obtained internal DCCs were 9.33 × 10^−5^ and 8.82 × 10^−5^ µGy·h^−1^/Bq·kg^−1^·FW for frond and root, respectively, and external DCCs were 5.58 × 10^−4^ and 5.63 × 10^−4^ µGy·h^−1^/Bq·kg^−1^·FW.

Since at the start of the experiment, no ^90^Sr is taken up yet by the plants, a time dependent dose rate approach was designed with the following assumptions: (i) The uptake ratio of ^90^Sr for both frond and root were given by their CR_FW_, which reached equilibrium phase at harvest time; (ii) The rate of uptake for all tested activity concentrations followed the same equation as obtained by the time course illustrated by Figure 1. Subsequently, the dose rate for fronds and roots (µGy·h^−1^) is given by Equations (1) and (2), respectively:

(1) $$\begin{array}{ll} {}[dose\;rate_{frond} & =\frac{1}{t}\int_{0}^{t}D_{total\;frond}\left(t\right)dt\\ & =AC_{m}\;\times \;DCC_{int\;frond}\;\times \;\int_{0}^{t}\;{\text{CR}}_{\text{FW}\;max\;frond}\left(1-e^{-\beta t}\right)\;dt+(AC_{m}\\ & \times DCC_{ext\;frond})/2\;] \end{array}$$

(2) $$\begin{array}{ll} {}[dose\;rate_{root}= & \frac{1}{t}\int_{0}^{t}D_{total\;root}\left(t\right)dt\\ & =AC_{m}\;\times \;DCC_{int\;root}\;\times \int_{0}^{t}\;{\text{CR}}_{\text{FW}\;max\;root}\left(1-e^{-\beta t}\right)\;dt\;+AC_{m}\;\times DCC_{ext\;root}\;] \end{array}$$

where *D_total_* is the total dose (µGy), *AC_m_* is the activity concentration measured in medium (Bq·L^−1^), *DCC_int organ_* and *DCC_ext organ_* are the internal and external dose conversion coefficients, respectively, for root or frond, unweighted for radiation quality (µGy·h^−1^/Bq·kg^−1^·FW), *CR_max organ_* is the organ specific CR_FW_ at equilibrium phase (Bq·kg^−1^·FW/Bq·L^−1^). β is the slope of the fit from the 7-day uptake experiment given by Figure 1 and *t* is the time of the exposure experiment which is set at 7 days. The *DCC_ext frond_* was divided by two since *L. minor* floats on water surface and therefore, only half of the fronds are surrounded by contaminated liquid medium. The total dose rate of the whole plant was calculated as the cumulative dose rate from frond and root separately, including organ-sized correction factors. The correction factors were the average fresh weight ratios for fronds and roots for each dose rate condition; (iii) Since *L. minor* exhibits an exponential growth with an average reproduction rate of 0.45 ± 0.02 fronds per day [64,65], the present experiment exposes approximately three generations of *L. minor* plants during this seven days treatment, meaning that not all plants were equally exposed with respect to exposure time. However, since the offspring of contaminated organisms are also subjected to equal dose rates, this dynamic approach considers the total population of *L. minor* as one organism with a treatment time of seven days, as in accordance with the OECD guidelines for the evaluation of chemical toxicants; (iv) ^90^Sr radionuclides were distributed homogeneously in both organs. However, since the morphologic dimensions of fronds and roots of *L. minor* fall within the range of the path length of a β-particle, this effect can be considered as negligible in this dosimetric approach.

### 4.4. Plant Growth

Growth parameters, in terms of relative frond number, relative frond area and fresh weight were analyzed according to OECD guidelines [33]. Images for determination of average specific growth rate and average specific frond area were taken at Day 0 and 7, and analyzed with ImageJ open source software (version 1.43) [63]. Fresh weight and dry weight for fronds and roots was determined as explained above. The average specific growth rate for the considered endpoint was calculated according to the OECD guidelines 221 [33]. Percentage growth inhibition was subsequently calculated for each test concentration; the average doubling time for frond number in non-irradiated controls was 1.74 ± 0.02, achieving the validity criterion of the experiment of ≤2.5 days as stated by the OECD guidelines. The pH in all test solutions remained constant at pH 5.5 ± 0.5. There were 9 replicates for each ^90^Sr treatment condition and control.

### 4.5. Antioxidative Enzyme Activities

Frozen plant tissue (50–80 mg·FW) was homogenized under frozen conditions with liquid nitrogen using two tungsten carbide beads (Qiagen, Venlo, The Netherlands) of 3-mm diameter in a Retsch Mixer Mill MM400 at 30 Hz for 3 min after a spatula of insoluble polyvinylpyrrolidone (PVP) was added. Hereafter, 400 µL ice-cold 0.1 M Tris-HCl buffer (pH 7.8) containing 1 mM EDTA and 1 mM dithiothreitol was added to the frozen homogenized tissue, the mixture was vortexed and subsequently centrifuged at 20,000× *g* at 4 °C for 10 min. The supernatant was kept on ice and used freshly for determination of enzyme capacities. All measurements were performed at room temperature (20–22 °C) based on spectrophotometric assays, as previously described [66]. Briefly, The capacity of CAT was analyzed by adding 10 μL of supernatants with 190 μL of a 49 mM H_2_O_2_ solution. The absorbance at 240 nm was monitored kinetically. To determine the GPX capacity, 150 μL 0.1 M phosphate buffer, 10 μL sample extract and 40 μL guajacol mastermix, consisting of 90 mM guaiacol and 163 mM H_2_O_2_ mixed on a 1:1 ratio were added in each well of a plastic 96-well plate. Subsequently, the appearance of tetraguajacol was monitored kinetically at 436 nm. SPX capacity was assessed in 96-well UV-plates. In each well, 155 μL 0.1 TRIS (pH 7.5), 20 μL 98 mM H_2_O_2_, 20 μL plant extract and 5 μL syringaldazine (SAZ) were added. The appearance of oxidized SAZ was monitored kinetically at 530 nm. For the determination of the GR capacity, 165 μL TRIS-EDTA buffer (0.1 M TRIS; 1 mM Na_2_-EDTA) (pH 8), 7 μL GR mastermix (1:1 mix of 82 mM GSSG and 6 mM NADPH) and 28 μL were added in each well of a 96-well UV-plate. The decrease of NADPH, used for the reduction of GSSG, was followed kinetically at 340 nm. The APX capacity was determined in UV-cuvettes using the Ultrospec 2000 UV/VIS Spectrophotometer (Pharmacia Biotech, Diegem, Belgium). In each cuvette, 665 μL HEPES-EDTA (0.1 M HEPES; 1 mM EDTA) buffer, 100 μL 30 mM Na-ascorbate, 35 μL 196 mM H_2_O_2_ and 200 μL sample extract were added. Subsequently, the appearance of dehydroascorbate (DHA) was measured spectrophotometrically at 298 nm. The activity of SOD was measured on the Ultrospec 2000 UV/VIS Spectrophotometer (Pharmacia Biotech) in plastic cuvettes. In each cuvette, 580 μL KH2PO4 buffer (pH 7.8), 100 μL 1 mM EDTA, 100 μL 0.5 mM xanthine, 100 μL 0.1 mM cytochrome C, 100 μL extract and 20 μL xanthine oxidase was added. The extent of cytochrome C reduction (measured at 550 nm) was compared to this in a blank, not containing any sample extract. Then, the relative inhibition of cytochrome C reduction by the sample extract was calculated as a measure of SOD capacity. At least 3 biological replicas were analyzed in triplicate (technical replicates). Statistical analysis was performed on the measured data before expressing them relative to controls.

### 4.6. Metabolite Measurements

Oxidized and reduced forms of ascorbate and glutathione were measured spectrophotometrically using a plate-reader assay, as described by [67]. Frozen plant tissue (50–80 mg·FW) was homogenized under frozen conditions using two tungsten carbide beads (Qiagen) of 3-mm diameter in a Retsch Mixer Mill MM400 at 30 Hz for 3 min in 600 μL of 200 mM HCl. After centrifugation (15 min, 13,000 rpm, 4 °C), 30 μL 200 mM NaH_2_PO_4_ (pH 5.6) was added to 300 μL of the supernatant. Subsequently, the pH of all samples was adjusted to 4.5 using 200 mM NaOH. For the measurement of total AsA, samples were incubated with 25 μM DTT and 120 mM NaH_2_PO_4_ (pH 7.5) for 15 min at 20 °C to fully reduce the AsA pool. Subsequently, the pH of the samples was adjusted to pH 5.5, the optimal pH for ascorbate oxidase, using 200 mM HCl. Further, all measurements were performed as described by Queval and Noctor [54]. Oxidized AsA and reduced GSH were calculated as the difference between total and reduced AsA and total and oxidized GSH, respectively. Furthermore, all measurements were performed as described by Queval and Noctor [67]. At least 3 biological replicas were analzed in triplicate (technical replicates). Statistical analysis was performed on the measured data before expressing them relative to controls.

### 4.7. Statistical Analysis

All data have been presented as mean values ± standard error (SE). Statistical analysis was performed with the open-source software package R (R i386 2.15.5; R Foundation for Statistical Computing, Vienna, Austria). Normal distribution was tested with a Shapiro–Wilk test, Barlett’s test was used to test for homoscedasticity. To identify any statistical differences between treatments, a one-way ANOVA was performed. When significant differences (*p*-value < 0.05) were found, a Tukey *post hoc* test was applied to further discriminate between significantly different groups. Student’s unpaired two-tailed *t*-test was used for single comparisons. The dose response curves were modeled using the three parameter log-logistic drm equation from the drc package available in the software package R (*p*-value < 0.05) [68]. Maximal growth reduction was fixed to 100% and the slope and EDR_50_ parameters were −0.99 and 238 for frond area, −1.02 and 391 for frond number, −0.96 and 509 for frond fresh weight, −0.92 and 732 for frond fresh weight, −0.67 and 39 for root fresh weight and −0.5 and 51 for root dry weight in the equation model.

## 5. Conclusions

In conclusion, our data demonstrated for the first time that β-radiation provokes a negative growth in *L. minor* plants. Submerged roots of *L. minor* plants were more radiosensitive to β-radiation compared to the floating fronds. All considered growth related endpoints showed a negative growth at the highest tested dose rate, although only a mild stimulation of the antioxidative defense system was observed. *L. minor* plants relied mainly on catalase to counteract the ROS generation following β-radiation, although some compounds of the ASC-GSH cycle were also activated, as they possessed higher levels of GSH at the highest tested dose rate level. The regulation of these antioxidative enzymes at the transcriptional level might be crucial in further examinations to support the observed biochemical changes.
